# Metabolite Profiling of Young Safflower (*Carthamus tinctorius* L.) Shoots at Different Harvest Times

**DOI:** 10.3390/ijms272110029

**Published:** 2026-11-09

**Authors:** Joanna Kapusta-Duch, Barbara Kusznierewicz, Barbara Borczak, Elżbieta Jędrszczyk, Teresa Leszczyńska

**Affiliations:** 1 Department of Human Nutrition and Dietetics, Faculty of Food Technology, University of Agriculture in Krakow, Al. Mickiewicza 21, 31-120 Krakow, Poland; barbara.borczak@urk.edu.pl (B.B.); teresa.leszczynska@urk.edu.pl (T.L.); 2 Department Chemistry, Technology and Biotechnology of Food, Faculty of Chemistry, Gdańsk University of Technology, 11/12 G. Narutowicza St., 80-233 Gdanskń, Poland; barbara.kusznierewicz@pg.edu.pl; 3 Department of Horticulture, Faculty of Biotechnology and Horticulture, University of Agriculture in Krakow, Al. Mickiewicza 21, 31-120 Krakow, Poland; elzbieta.jedrszczyk@urk.edu.pl

**Keywords:** young shoots, *Carthamus tinctorius* L., harvest time, metabolite profiling, LC–HRMS, antioxidant activity, phenolic compounds

## Abstract

Safflower (*Carthamus tinctorius* L.) is an oilseed plant species within the *Asteraceae* family. It is cultivated on a large scale and is widely used in traditional herbal medicine. However, information on changes in the metabolite profiles of young safflower shoots at different harvest times remains limited. Comprehensive metabolite profiling of *Carthamus tinctorius* L. young shoots harvested at 11, 18, and 21 days after sowing was performed using LC–Q-Orbitrap HRMS. A total of 76 compounds were tentatively identified, representing several major phytochemical groups, including amino acids and related compounds, carbohydrates, other glycosides, phenylpropanoid glycosides, apocarotenoids, hydroxycinnamic acid amides, flavonoids, lignans, and lipids. Exploratory principal component analysis and hierarchical clustering revealed clear differences in metabolite profiles among the three harvest times. Most of the semi-quantified metabolites showed lower contents at the later harvest times, although distinct patterns were observed for individual metabolites. Post-column ABTS profiling indicated that antioxidant activity was predominantly associated with quercetin and luteolin derivatives. Overall, the results demonstrate marked, harvest-time-associated differences in the phytochemical profiles of young safflower shoots.

## 1. Introduction

*Carthamus tinctorius* L.—an oilseed crop—commonly known as safflower (or false saffron), is a member of the *Asteraceae* family. It is an annual herbaceous plant, cultivated primarily for its seeds, meal and flowers [1,2]. Research undertaken to date on the traditional and modern uses of *Carthamus tinctorius L.* indicates that this plant is of great economic importance [3]. Numerous clinical and laboratory studies have focused on the therapeutic effects and mechanisms of action of *Carthamus tinctorius L.*, and their findings point to the plant’s broad spectrum of biological activity and its clinical efficacy in the treatment of various conditions [4]. Safflower (*Carthamus tinctorius* L.) extracts have demonstrated considerable therapeutic potential, driven by their robust antioxidant, anti-inflammatory, and apoptosis-modulating properties. Research confirms their beneficial impact on the cardiovascular system, exhibiting anticoagulant, hypotensive, as well as cardio- and vasoprotective activities. They also have protective and therapeutic effects across various other tissues and organs, including neuroprotective, hepatoprotective, and antidiabetic outcomes [5,6,7].

A significant range of chemical compounds are present in medicinal plants, the metabolite profiles of which are shaped by gene expression networks driven by time and environmental factors [8]. Therefore, understanding the factors that influence the accumulation of bioactive metabolites is important for characterising the phytochemical and potential functional properties of plant material. Omics approaches, including transcriptomics, proteomics, and metabolomics, provide complementary information on plant biochemical processes [9]. Among these approaches, metabolomic profiling enables direct assessment of changes in a broad range of low-molecular-weight metabolites and can therefore provide valuable insight into changes in phytochemical composition associated with plant development [10].

Metabolomics is a highly effective approach to comprehensively evaluate the quality of medicinal plants, to develop new drugs from existing resources and to find new medicinal materials from latent resources. This makes it a critical part of the process when developing new drugs from traditional medicinal resources [11].

Microgreens, also referred to as microleaves, are young edible shoots of vegetables, herbs, grains, and other plant species, typically harvested after cotyledon expansion and around the emergence of the first true leaves [12,13,14,15]. Unlike sprouts, which are generally consumed together with the seed and root, microgreens are harvested by cutting the above-ground portion of the plant [14,15]. Depending on the species and cultivation conditions, microgreens are generally harvested within a few weeks after germination [12,14]. Young edible shoots collected at later developmental stages may extend beyond the conventional definition of microgreens. Therefore, in the present study, which included safflower plants harvested at 11, 18, and 21 days after sowing, the more general term “young shoots” is used throughout the manuscript, consistent with terminology previously applied to safflower harvested at different stages of vegetation [13].

Young edible shoots have attracted increasing interest as sources of phytochemicals and other nutritionally relevant constituents [13,14,15,16,17,18]. Their phytochemical composition may vary considerably with plant species and developmental or harvesting stage, making harvest time an important factor affecting their nutritional and functional characteristics [12,13].

The phytochemistry of *Carthamus tinctorius* L. at the fully mature stage has been extensively investigated. However, information on the metabolite profiles of young safflower shoots collected at different harvest times remains limited. Therefore, the aim of this study was to characterize the metabolite profiles of young safflower shoots harvested at 11, 18, and 21 days after sowing using LC–HRMS and to compare their phytochemical profiles using exploratory multivariate analysis. In addition, selected major metabolites were semi-quantified, and compounds contributing to the antioxidant activity of the extracts were evaluated using post-column ABTS profiling.

## 2. Results

### 2.1. Identification of Major Metabolites

**Figure 1:**
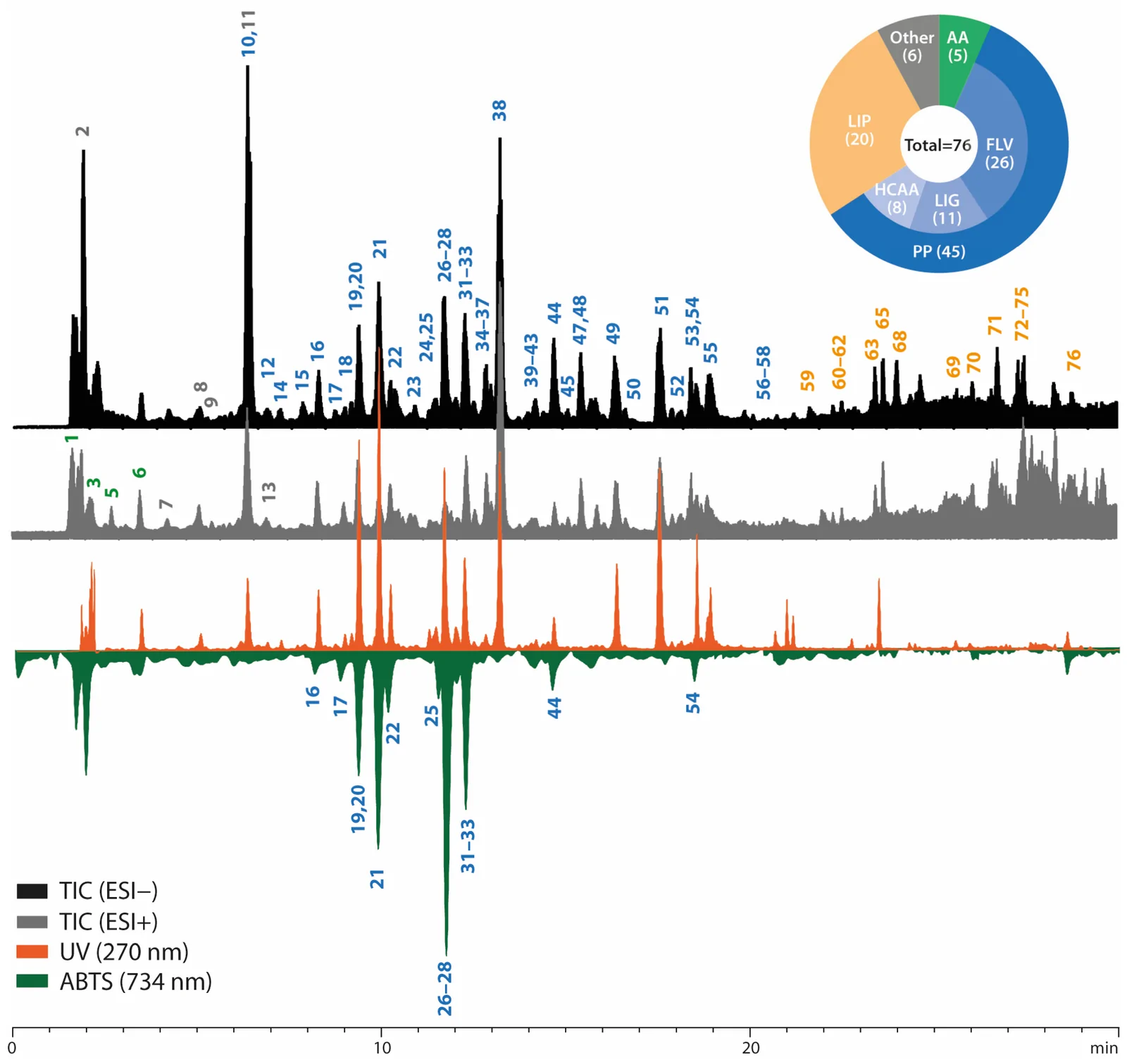
Representative LC-HRMS, UV, and post-column ABTS chromatographic profiles of *Carthamus tinctorius* L. young shoot extracts. The overlaid chromatograms include TIC acquired in negative (ESI−) and positive (ESI+) ionization modes, UV detection at 270 nm, and ABTS radical scavenging detection at 734 nm. Peak numbers correspond to the metabolites listed in Table 1. The inset pie chart summarizes the distribution of the identified metabolites among the major chemical classes: amino acids (AAs), lipids (LIPs), and phenolic compounds (PPs), including flavonoids (FLVs), lignans (LIGs), and hydroxycinnamic acid amides (HCAAs).

The comprehensive metabolite profiling of *Carthamus tinctorius* L. young shoots was performed using LC-HRMS/MS in both positive and negative ionization modes, complemented by UV detection at 270 nm and post-column ABTS radical scavenging detection at 734 nm. Representative chromatographic profiles are presented in Figure 1, where the peak numbering corresponds to the compounds tentatively identified in Table 1. The inset pie chart illustrates the distribution of the detected metabolites among the major chemical classes. Flavonoids constituted the largest group of annotated metabolites (26 compounds), followed by lipids (20 compounds), while the remaining metabolites represented amino acids and related compounds, carbohydrates, other glycosides, phenylpropanoid glycosides, apocarotenoids, hydroxycinnamic acid amides, and lignans.

Based on accurate mass measurements, molecular formula prediction, characteristic MS/MS fragmentation patterns, comparison with literature data, and database searches, a total of 76 metabolites were tentatively identified (Table 1). The identified compounds belonged to several major phytochemical groups, including amino acids and related compounds, carbohydrates, other glycosides, phenylpropanoid glycosides, apocarotenoids, hydroxycinnamic acid amides, flavonoids, lignans, and lipids.

The early-eluting compounds were mainly represented by amino acids and highly polar metabolites. Spermidine was identified based on characteristic polyamine-derived fragments at *m*/*z* 72.0816, 84.0815, and 112.1124, while phenylalanine and tryptophan produced diagnostic phenethylamine-, tropylium-, and indole-related product ions. The disaccharide was tentatively assigned from its molecular formula and the occurrence of characteristic carbohydrate-derived fragments, including ions at *m*/*z* 179.0550 and 161.0448.

Among the glycosidic constituents, rengyoside B was classified as another glycoside, whereas syringin and dihydrosyringin were identified as phenylpropanoid glycosides. Their annotation was supported by characteristic product ions corresponding to rengyoxide, sinapyl alcohol, and dihydrosinapyl alcohol moieties. In addition, thalictoside was tentatively identified as a nitroaromatic glycoside based on the presence of diagnostic nitroethyl-derived fragments and the aglycone ion corresponding to 4-(2-nitroethyl)phenol.

Flavonoids constituted the largest group of identified specialized metabolites (Figure 1). Numerous glycosides and acylated derivatives of apigenin, luteolin, quercetin, and acacetin were detected. *O*-glycosylated flavonoids were characterized by the formation of abundant aglycone ions at *m*/*z* 269.0456 (apigenin), 285.0404 (luteolin), 301.0354 (quercetin), and 283.0613 (acacetin). In contrast, *C*-glycosyl flavones such as apigenin-*C*-hexoside, apigenin-*C*-dihexoside, and apigenin-*C*-pentosyl-*C*-hexoside exhibited characteristic cross-ring cleavage fragments, which enabled differentiation from their *O*-glycosylated counterparts. Acetylated and malonylated flavonoid derivatives were further recognized by neutral losses associated with the corresponding acyl substituents.

Hydroxycinnamic acid amides were represented by N-coumaroylserotonin and N-feruloylserotonin together with their glycosylated and malonylated derivatives. Their identification was based on characteristic aglycone ions at *m*/*z* 321.1246 and 351.1352, respectively, accompanied by serotonin-derived fragments as well as diagnostic coumaroyl- and feruloyl-derived product ions.

Lignans represented another abundant group of metabolites detected in safflower young shoots. The identified compounds included derivatives of olivil, matairesinol, syringaresinol, trachelogenin, and tracheloside. Their annotation was supported by characteristic product ions formed following the loss of sugar and/or acyl moieties. Diagnostic ions corresponding to matairesinol (*m*/*z* 357.1346), trachelogenin (*m*/*z* 387.1453), hydrated trachelogenin (*m*/*z* 405.1553), and syringaresinol (*m*/*z* 417.1559) supported the annotation of numerous glycosylated and acylated lignan derivatives. Notably, lignans constituted one of the dominant subclasses of phenolic compounds detected in the analyzed young shoots (Figure 1).

Lipid profiling revealed the presence of free fatty acids, oxidized fatty acids, galactolipids, sulfolipids, phospholipids, and sphingolipids. Monogalactosyldiacylglycerols (MGDGs), monogalactosylmonoacylglycerols (MGMGs), digalactosyldiacylglycerols (DGDGs), digalactosylmonoacylglycerols (DGMGs), sulfoquinovosyldiacylglycerols (SQDGs), sulfoquinovosylmonoacylglycerols (SQMGs), phosphatidylethanolamine (PE), and hexosylceramide (HexCer) species were tentatively identified based on characteristic fatty-acyl fragments, galactosyl-derived ions, phosphoethanolamine fragments, and diagnostic sulfoquinovosyl product ions at *m*/*z* 225.007.

### 2.2. Comparative Analysis of Metabolite Profiles

**Figure 2:**
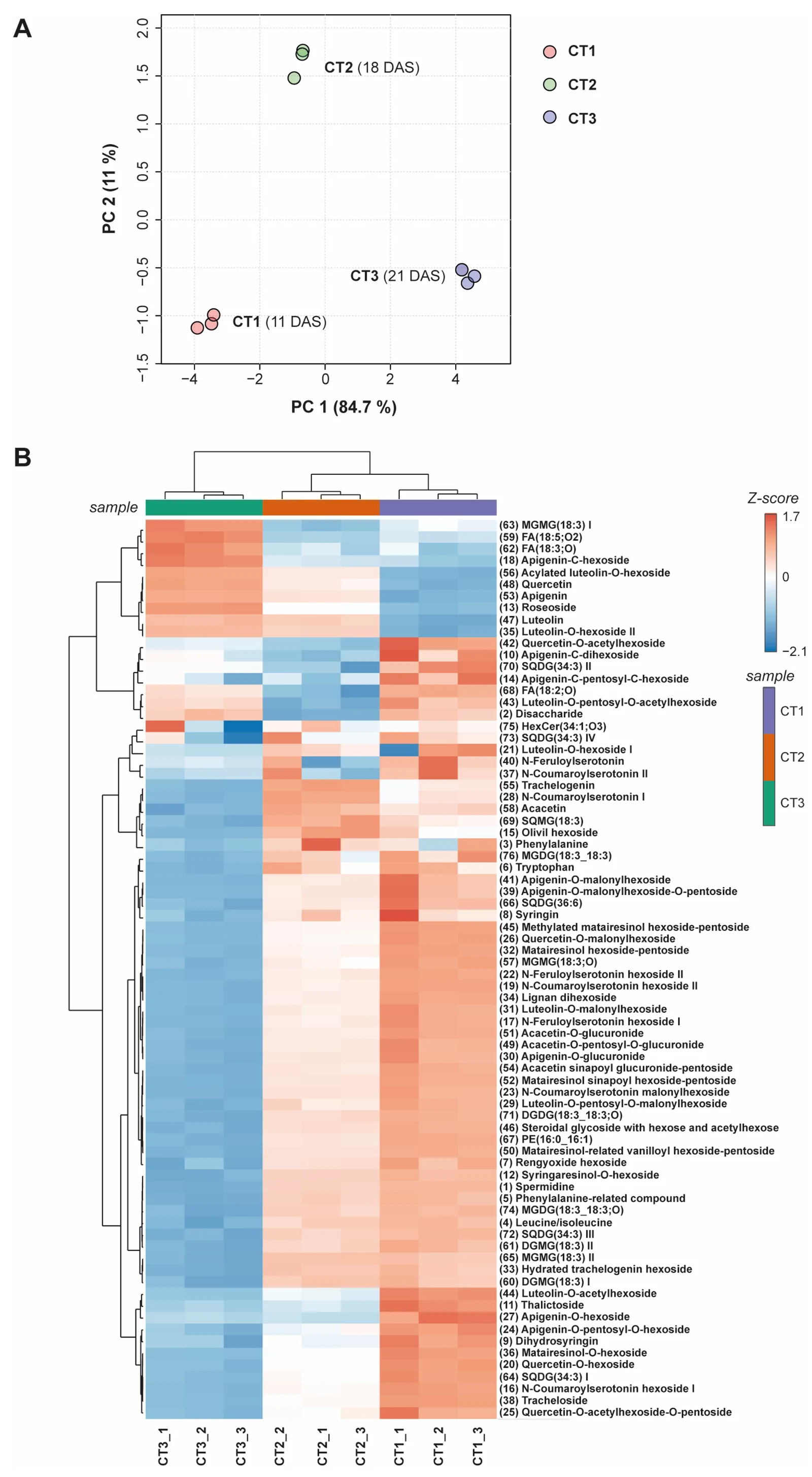
Comparative analysis of metabolite profiles in safflower young shoots harvested at 11 (CT1), 18 (CT2), and 21 (CT3) days after sowing (DAS). (**A**) Principal component analysis (PCA) score plot based on the peak areas of 76 annotated metabolites. Data were log10-transformed and Pareto-scaled prior to PCA. PC1 and PC2 explained 84.7% and 11.0% of the total variance, respectively. (**B**) Hierarchical clustering heatmap of the 76 annotated metabolites. Colors represent feature-wise standardized LC-HRMS peak-area values. Hierarchical clustering of samples and metabolites was performed using Euclidean distance and Ward’s linkage method. For each harvest time, three parallel extracts prepared from the same pooled lyophilized plant material are shown.

Principal component analysis (PCA) was performed using the peak areas of the 76 metabolites tentatively identified by LC-HRMS/MS (Table 1) to evaluate differences in the metabolite profiles of young safflower shoots harvested at 11, 18, and 21 days after sowing (DAS), designated as CT1, CT2, and CT3, respectively. Prior to PCA, the peak-area data were log10-transformed and Pareto-scaled. The first two principal components explained 95.7% of the total variance, with PC1 and PC2 accounting for 84.7% and 11.0% of the variance, respectively (Figure 2).

The PCA score plot revealed a clear separation of the CT1, CT2, and CT3 samples, indicating marked differences in their metabolite profiles. The three parallel extracts prepared from each pooled lyophilized sample clustered closely together, indicating good reproducibility of the extraction and analytical procedure. The greatest discrimination was observed along PC1, which separated CT1 and CT3 samples located on opposite sides of the score plot, suggesting marked differences in the relative abundance patterns of the annotated metabolites between these harvest times. In contrast, CT2 samples occupied an intermediate position along PC1 but were clearly distinguished from both CT1 and CT3 along PC2, indicating a distinct metabolite profile at 18 DAS.

To further examine metabolite-specific patterns across the three harvest times, hierarchical clustering analysis was performed and visualized as a heatmap based on the feature-wise standardized peak-area data of the 76 annotated metabolites (Figure 2B). The clustering pattern was consistent with the PCA results: the three parallel extracts corresponding to each harvest time clustered together, while CT1, CT2, and CT3 formed distinct sample clusters. A large group of metabolites showed progressively lower relative signals from CT1 to CT3, including numerous flavonoids, lignans, hydroxycinnamic acid amides, and lipid derivatives.

In contrast, a smaller cluster of metabolites exhibited a different pattern, with the highest relative signals observed in CT3 samples. This group comprised several flavonoids and oxidized lipid derivatives.

A third group comprised metabolites showing the highest relative signals in CT2 samples, including representatives of flavonoids, lignans, lipid derivatives, and hydroxycinnamic acid amides. This distinct pattern was consistent with the separation of CT2 samples from CT1 and CT3 observed along PC2 in the PCA score plot.

### 2.3. Semi-Quantitative Analysis of Major Metabolites and Post-Column ABTS Antioxidant Profiling

Semi-quantitative analysis revealed differences in the contents of major metabolites among safflower young shoots harvested at 11, 18, and 21 DAS (Table 2). Among the individually resolved metabolites included in the semi-quantitative analysis, luteolin-O-hexoside showed the highest content at all three harvest times, followed by acacetin-O-glucuronide. For incompletely resolved chromatographic peaks, the reported values were treated as approximate estimates of the corresponding unresolved signals and were not used for compound-level abundance ranking. Most of the semi-quantified signals showed their highest values in CT1 and lower values at the later harvest times, consistent with the overall patterns observed in the PCA and hierarchical clustering analyses.

The post-column ABTS assay showed that antioxidant activity was associated predominantly with quercetin and luteolin derivatives, whereas lignans and most acacetin derivatives did not produce detectable negative peaks in the ABTS chromatograms (Figure 1). Interestingly, acacetin sinapoyl glucuronide-pentoside exhibited a detectable antioxidant response, in contrast to the remaining acacetin derivatives. The hydroxycinnamic acid amide N-coumaroylserotonin hexoside also showed a detectable antioxidant response. Conversely, the identified lignans did not produce detectable antioxidant responses under the applied post-column ABTS conditions.

## 3. Discussion

Safflower is characterised by a complex chemical profile, featuring a wide spectrum of compounds, with flavonoids exhibiting particularly notable pharmacological activity. It is evident that these compounds find extensive application in the pharmaceutical, food, and cosmetic industries; thus, safflower is positioned as a promising candidate for development and application in the treatment of various diseases.

In this study, 76 metabolites were tentatively identified, representing several major phytochemical groups, including amino acids and related compounds, carbohydrates, other glycosides, phenylpropanoid glycosides, apocarotenoids, hydroxycinnamic acid amides, flavonoids, lignans and lipids. The available literature contains a great deal of information on the chemical composition of fully mature safflower flowers and seeds. However, there is a lack of detailed data specifying the chemical profile of young shoots. To date, over 200 compounds have been isolated from mature safflower. These compounds can be divided into the following subgroups: flavonoids, fatty acids, phenylethanol glycosides, coumarins, steroids and polysaccharides [21].

The evident therapeutic effect is linked to the chemical composition and pharmacological properties of the safflower. In recent years, a significant number of bioactive compounds have been identified using a variety of analytical methods. These methods include liquid chromatography coupled with mass spectrometry (LC-MS), gas chromatography coupled with mass spectrometry (GC-MS), high-throughput metabolic profiling and nuclear magnetic resonance (NMR) [1,2,3,5,21,22,23,24,25,26,27].

Safflower is an oilseed crop, with oil composition influenced by variety and environmental factors. The oil comprises ~90% unsaturated fatty acids, predominantly linoleic (71–75%), oleic (16–20%), palmitic (6–8%), and stearic (2–3%) acids [24]. It serves as a rich source of tocopherols, phytosterols, phospholipids, and carotenoids [28], with a notable phytosterol profile including β-sitosterol, campesterol, avenasterol, stigmasterol, stigmastenol, and brassicasterol [29]. The plant also supplies essential nutrients, including sugars, minerals, and a comprehensive profile of amino acids [30]. Additionally, safflower seeds contain 13.17–17.36% protein [31] and 11–22% fibre [32], while bioactive triterpenoid saponins are present in the roots [33]. The increase in oxylipin levels during the development of young shoots is directly linked to the physiological and adaptive processes of seedlings. During photomorphogenesis, cotyledon development and exposure to light, biological membranes undergo remodelling and lipoxygenases (LOXs) are activated. These enzymes oxidise polyunsaturated fatty acids, primarily linoleic and α-linolenic acids. The resulting oxylipins (including jasmonoids) act as signalling molecules that regulate plant growth and its responses to biotic and abiotic stresses, such as light or mechanical resistance from the growing medium. Furthermore, intensive cell division and elongation require constant remodelling of membrane lipids. This process generates free fatty acids, which in turn serve as a substrate for the biosynthesis of oxylipins [34,35]. Other researchers also point to a dynamic change in the phytochemical profile—compounds characteristic of the seeds are gradually disappearing, whilst the proportion of metabolites associated with photosynthesis and the stomatal apparatus (e.g., O-glycosylated flavonoids that protect against UV radiation) is increasing [36,37,38].

Flowers of *Carthamus tinctorius* L. yield diverse active compounds, including polyacetylenes, lignans, phenolic acids, polysaccharides, sterols, and flavonoids [7]. Safflower uniquely produces quinochalcone C-glycoside pigments, encompassing yellow derivatives (e.g., hydroxysafflor A, safflor B, and tinctoramine) and the primary red pigment, carthamin [2,4,39,40]. The absence or inability to detect quinochalcones (such as cartamine or hydroxysafflor yellow A) in the samples tested can be explained in several ways. The biosynthesis of quinochalcones in *Carthamus tinctorius* L. exhibits a high degree of organ-specific and ontogenetic specificity, and their accumulation is concentrated primarily in the generative phase within the corolla petals. In vegetative tissues, including at the young shoot stage, the metabolic pathways responsible for the mass production of these pigments are not yet fully activated. The presence of quinochalcone analogues in seedlings, as detected using highly sensitive analytical techniques (e.g., HPLC-MS/MS), is typically at the limit of quantification (LOQ). Furthermore, the profile and concentration of these metabolites can vary significantly due to genotypic differences. Modern oil-producing safflower varieties are characterised by a substantial reduction or repression of the biosynthetic pathways involved in pigment synthesis, in contrast to traditional commercial lines [19,41].

Safflower flowers also contain other flavonoids, predominantly flavones and flavonols (such as glycosides of kaempferol, quercetin, acacetin, and apigenin), alongside lignans (methylsyringin, ethylsyringin, tracheloside), *p*-coumaric acid derivatives, polyacetylenes, and spermidine derivatives [1,4,40]. The high content of phytochemicals is undoubtedly the most valuable nutritional feature for health-conscious consumers. It is a well-known fact that sprouting increases the phytochemical content of sprouts and young shoots in comparison with their non-sprouted counterparts. However, the scale, extent and direction of this change vary depending on the species, variety and growing conditions [16,42,43]. In this study, a statistically significant decrease in the content of selected polyphenols was observed at these very early harvest stages (i.e., on days 11, 18 and 21). The decline in the concentration of selected phenolic compounds during the development of young shoots is most likely due to the interaction of two main mechanisms. The first of these is the so-called growth dilution effect. The early stages of seedling development are characterised by rapid increases in fresh and dry weight, which are associated with intensive water uptake and the synthesis of structural components such as cellulose and hemicellulose. The second mechanism is linked to metabolic reprogramming, which is the transition from the seed stage, in which metabolites primarily serve storage and protective functions, to the stage of active photosynthesis. For instance, serotonin conjugates, matairesinol and tracheloside are substances that are characteristic of *Carthamus tinctorius* seeds. During the germination process, they undergo a series of enzymatic breakdowns and are transported to developing tissues. In these tissues, they serve a number of functions, including providing a source of nitrogen (in the case of serotonin conjugates) and contributing to the building process [44,45]. The enhanced digestibility of sprouts and young shoots is attributable to the enzymatic activity that occurs during germination, which facilitates the breakdown of macromolecules into more straightforward compounds, such as amino acids, fatty acids and simple sugars. The germination process also reduces the content of anti-nutritional compounds, including trypsin inhibitors, which interfere with protein digestion, and phytates, which hinder the absorption of minerals [15]. It is well-documented that young shoots possess a range of health-promoting properties. The results obtained are consistent with the general trend observed in studies on the metabolomics of young shoots of various species (e.g., from the *Brassicaceae*, *Fabaceae* or *Asteraceae* families). As confirmed by the majority of publications, the concentration of phenolic compounds per gram of dry/fresh weight is higher in the youngest seedlings (sprouts/young shoots) than in mature plants [46,47,48,49].

This is not unexpected, given that phytochemicals frequently exhibit synergistic effects when present in combination [50]. For this reason, young shoots should not be regarded solely as a source of individual phytochemicals for pharmaceutical extraction and purification in medicinal preparations. Instead, they should be regarded as ready-to-eat functional foods or nutraceuticals. Conversely, the profile of secondary plant metabolites can vary significantly due to the phenomenon of chemical polymorphism, resulting in the presence of multiple chemotypes within the same species [51]. Chemotypes are defined as the same species/subspecies/varieties of an organism containing different types of secondary metabolites or the same secondary metabolites in varying quantities [52]. It should be emphasised that an analysis based on a single variety and a single growing season limits the possibility of directly extrapolating the results to a wider scale.

A study by Dereje et al. [12] investigated the effect of harvest time on the phytochemical profile of microgreens from the *Brassicaceae* family. The study found that harvest time is a key factor determining phytochemical content. The microgreens used for laboratory analysis were harvested on days 7, 11, 15 and 19 after sowing, in order to capture likely rapid, dynamic changes in their phytochemical metabolism. The authors observed that, in the case of rocket microgreens, extending the harvesting period promoted the accumulation of chlorophyll, carotenoids, total phenolics, flavonoids and anthocyanins, all of which contributed to an increase in antioxidant capacity. In contrast, microgreens of cress exhibited a selective response, with increases observed mainly in chlorophyll and anthocyanins. This indicates that an extended harvest period does not enhance phytochemical richness to the same extent in all species, even within the same botanical family [12].

In a previous publication by the authors of this paper, the basic composition, vitamin C content, total polyphenols and total carotenoids in young shoots of safflower, black cumin and camelina were determined. Furthermore, the antioxidant activity of these young shoots at different stages of development was investigated [13]. The first harvest was carried out when the plants had cotyledons and their first true leaf, and the second harvest when each plant had 1–2 leaves. As Kapusta-Duch et al. [13] observed in their study, young shoots in the phase of intensive growth were characterised by very high levels of the analysed components and antioxidant properties. These levels varied depending on the harvest date and plant species. It was found that young shoots from the first harvest had significantly higher levels of protein, fat and certain minerals than shoots from the second harvest. Research has shown that the antioxidant properties of young shoots generally increase with their degree of maturity. The research team, led by Zhou et al. [51], investigated the manner in which sucrose stimulation enhances the anti-glycaemic effect in safflower sprouts. The findings of this study can be applied to plants at the microgreen stage. Zhou et al. [51] found that luteolin-7 glucoside accumulates in the cotyledons; it therefore follows that the optimal time to harvest microgreens for maximum content of this valuable flavonoid is when the cotyledons are fully developed. Yadav et al. [52] concluded that the heightened concentrations of phenolic antioxidants in mature phases may be attributable to the synthesis of these compounds, as plant defence molecules are known to increase via the activation of the phenylpropanoid pathway. This differs from the general pattern observed in the present study, in which most of the semi-quantified metabolites showed lower contents at the later harvest times. However, because the samples were collected at successive developmental stages under natural greenhouse light conditions, the observed differences cannot be attributed exclusively to plant developmental stage. They should therefore be interpreted as changes in metabolite profiles associated with successive harvest times during the early growth of safflower. One potential reason for these discrepancies could be the low specificity of the Folin–Ciocalteu (F-C) assay, which measures total reducing capacity rather than specific phenolic compounds. Non-phenolic reducing substances present in the plant matrix—such as ascorbic acid, sugars, or amino acids—can interfere with the assay, leading to an overestimation of the total phenolic content compared to the targeted LC-MS/MS quantification [53]. 

In the study, Kapusta-Duch et al. [13] found that all varieties of young shoots from the second harvest exhibited significantly higher levels of vitamin C, total carotenoids and total polyphenols in comparison with young shoots from the first harvest. The antioxidant activity of the young shoots exhibited statistically significant variation depending on the time of harvest and the species of young shoot. Karamać et al. [54] analysed the polyphenol profile of the camelina plant (*Camelina sativa* L.), collected at five different phases of development. These phases ranged from the vegetative stage to the stage of mature seed pods. The total content of polyphenols in the camelina extracts ranged from 49.2 to 59.1 mg GAE/g FW, and the discrepancies were not statistically significant for the various growth stages examined.

The results cited in this paragraph, obtained by various authors, confirm the ambiguous and varied trends in changes to total polyphenol content and antioxidant activity in young, developing plant organisms.

The post-column ABTS profiling revealed marked differences in antioxidant response among the identified phenolic compounds. Quercetin and luteolin derivatives produced detectable negative peaks in the ABTS chromatograms, which is consistent with established structure–activity relationships of flavonoids. The catechol moiety in the B-ring is considered an important structural feature contributing to radical-scavenging activity, while the additional 3-hydroxyl group present in quercetin may further enhance its antioxidant potential [55]. In contrast, most acacetin derivatives did not show detectable antioxidant responses under the applied conditions. This observation may be related to methylation of the 4′-hydroxyl group, as *O*-methylation of the catechol moiety has been shown to alter the radical-scavenging properties of flavonoids [56]. Interestingly, acacetin sinapoyl glucuronide-pentoside exhibited a detectable antioxidant response, suggesting that the sinapoyl moiety may contribute to the activity of this conjugate, which is consistent with the established radical-scavenging properties of sinapic acid and its derivatives [57]. N-Coumaroylserotonin hexoside also showed a detectable antioxidant response. Hydroxycinnamic acid amides of serotonin, including *p*-coumaroylserotonin, have previously been reported to exhibit antioxidant and radical-scavenging properties [58]. The identified lignans did not produce detectable responses in the post-column ABTS assay, indicating no detectable radical-scavenging response under the applied chromatographic conditions.

## 4. Materials and Methods

### 4.1. Plant Material

High-quality, wholesome seeds of safflower (*Carthamus tinctorius* L.) cv. *Saffire* were obtained from PlantiCo—Horticultural Breeding and Seed Production Zielonki Sp. z o.o. (Stare Babice, Poland; product code: KRF-AA-0001.00-KRS-N). Prior to sowing, seed quality—including germination capacity, purity, and moisture content—was laboratory-verified by the producer in accordance with International Seed Testing Association (ISTA) procedures. Sowing was carried out at the turn of May and June in 6 seed boxes (600 × 400 mm) filled with TS 2 peat substrate (Klasmann-Deilmann GmbH, Geeste, Germany; pH = 6.0), enriched with PG Mix fertilizer (2 g/L) and a surfactant. The fertiliser formulation was 2 g/L, and its composition was as follows: 16% phosphorus (P_2_O_5_), 14% total nitrogen (consisting of 5.5% N-NO_3_ and 8.5% N-NH_4_), 18% potassium (K_2_O), 19% sulfur (SO_3_) and 0.8% magnesium (MgO). The product was further enriched with micronutrients, including 0.20% molybdenum (Mo), 0.16% manganese (Mn), 0.12% copper (Cu), 0.09% iron (Fe), 0.04% zinc (Zn), and 0.03% boron (B). The sowing density was maintained at a range of 0.5–1.5 seeds per square centimetre. The cultivation was conducted in a greenhouse at the Faculty of Biotechnology and Horticulture, University of Agriculture in Krakow, under natural photoperiod conditions. The plants were exposed to ambient greenhouse light (i.e., ambient greenhouse solar radiation; 14 h light/10 h dark). The climate parameters were strictly controlled at 21 °C and 50–63% relative humidity during the day, shifting to 18 °C and 78–88% relative humidity during the night. The water used for daily irrigation was characterised by a pH of 7.6 and an electrical conductivity of 683 µS/cm at 25 °C. The total water hardness was 301 mg/dm^3^ while the oxidizability determined using potassium permanganate reached a value of 0.9 mg/dm^3^. Physical parameters indicated a very low turbidity not exceeding 0.1 NTU and a low color intensity of 5 mg/dm^3^. The mineral composition was dominated by calcium ions at a concentration of 101 mg/dm^3^, magnesium at 11.6 mg/dm^3^, and sodium at 21 mg/dm^3^. Among the anions, chlorides were recorded at 44.3 mg/dm^3^, sulfates at 48 mg/dm^3^, nitrates at 16.3 mg/dm^3^, and fluorides at a concentration of 0.08 mg/dm^3^. The content of ammonium ions and heavy metals, such as total iron, manganese, chromium, cadmium, copper, and nickel, remained at trace levels below 0.021 mg/dm^3^.

A daily observation regime was implemented, encompassing the monitoring of plant health. With the exception of watering, no other maintenance activities were required. Young shoots were harvested at three distinct developmental stages according to the BBCH scale [59].The first harvest was conducted 11 days after sowing at BBCH stage 11, characterized by fully expanded cotyledons and one unfolded true leaf. The second and third harvests occurred 18 days after sowing at BBCH stage 12, with two unfolded true leaves, and 21 days after sowing at BBCH stage 13, with three unfolded true leaves, respectively.

At each developmental stage, the above-ground portions of young plants were meticulously excised using sterile scissors. The fresh material was harvested, rinsed with tap water, cut into small pieces (1–2 cm), and then pooled to prepare representative fresh samples. In order to undergo the freeze-drying process, the remaining raw material was subjected to a thorough rinsing procedure, followed by the removal of excess moisture via a process of blotting on filter paper. Subsequently, the material underwent a mechanical crushing step, and was then frozen at a temperature of −22 °C. Lyophilization was performed using a Christ Alpha 1–4 unit (Osterode am Harz, Germany). The freeze-dried product was ground in a laboratory mill (Knifetec 1095 Sample Mill, Tecator, Höganäs, Sweden) equipped with titanium-niobium-coated blades to prevent heavy metal contamination, yielding a fine, homogeneous powder. Subsequently, the freeze-dried material was examined.

### 4.2. Analytical Methods

#### 4.2.1. Extract Preparation

The ground and homogenized samples (70 mg) were extracted with 1 mL of 70% ethanol aqueous solution and placed in an ultrasonic bath (POLSONIC, Warsaw, Poland) for 20 min at 25 °C. The samples were centrifuged (15,600× *g*, 20 min) and clear supernatants were collected. Three parallel extractions were performed independently from each pooled lyophilized sample.

#### 4.2.2. LC-Q-Orbitrap HRMS Analysis of Plant Extracts

Qualitative analyses were performed on a Dionex UltiMate 3000 UHPLC system (Thermo Fisher Scientific, San Jose, CA, USA). Separations employed Kinetex^®^ XB-C18 column (150 × 4.6 mm, 5 μm; Phenomenex, Torrance, CA, USA). The mobile phase consisted of water (A) and acetonitrile (B), each containing 0.1% (*v*/*v*) formic acid; flow rate 0.8 mL min^−1^. Injection volumes and gradients were as follows: *C. tinctorius*: 3 μL; 0 min, 10% B; 15 min, 30% B; 25 min, 100% B; and 30 min, 100% B.

The chromatographic system was coupled to a PDA detector (UV–Vis) and a Q Exactive™ Focus quadrupole-Orbitrap mass spectrometer (Thermo Fisher Scientific, Bremen, Germany) equipped with a HESI-II source. The HESI parameters in negative and positive ion acquisition mode were as follows: auxiliary gas flow rate, 15 arb; sheath gas flow rate, 35 arb; sweep gas flow rate, 3 arb; capillary temperature, 350 °C; spray voltage, 2.5 kV; S-lens RF level, 50; and heater temperature, 300 °C. The full MS scan was set as follows: resolution, 70,000 FWHM; AGC target, 2 × 10^5^; max inject time, 100 ms; and scan range, 120–1200 *m*/*z*.

The data-dependent MS2 parameters were as follows: resolution, 17,500 FWHM; isolation window, 3.0 *m*/*z*; normalized collision energy (NCE), 30; AGC target, 5 × 10^4^; maximum injection time, auto; loop count, 1; dynamic exclusion, auto; and isotope exclusion, enabled.

A pooled quality control (QC) sample was prepared by combining aliquots of all extracts. The nine sample extracts were analyzed in randomized injection order within a single analytical sequence. Three blank injections were performed at the beginning of the sequence. The pooled QC sample was injected before the first sample, after every three sample injections, and at the end of the sequence, resulting in four QC injections in total.

Raw mass spectrometric data were processed using Compound Discoverer software (version 3.3.3.200, Thermo Scientific, San Jose, CA, USA). Signal intensities were normalized by QC-based area correction, applying a minimum QC coverage threshold of 50% and a maximum QC relative standard deviation (RSD) of 30%. For the retained features, the median QC RSD before QC-based area correction was 14% in negative-ion mode and 7% in positive-ion mode, decreasing after correction to 4% and 2%, respectively. After correction, 98.9% of retained features in negative-ion mode and 99.3% in positive-ion mode showed QC RSD values ≤10%, and all retained features in both ionization modes showed QC RSD values ≤15%. Compound annotation was performed based on accurate mass measurements within a tolerance of ±5 ppm, retention time alignment (±0.2 min), and MS^2^ fragmentation characteristics. Compound annotation was performed by manual interpretation of accurate mass and MS^2^ fragmentation data and comparison with fragmentation patterns reported in the literature. Spectral library and database searches implemented in Compound Discoverer were used as supporting information where suitable matches were available. Accordingly, compound assignments should be regarded as tentative annotations rather than confirmed identifications, unless otherwise stated.

#### 4.2.3. Semi-Quantitative Analysis of Selected Metabolites by LC-PDA

Standard solutions were prepared by dissolving 1 mg of each reference compound in 1 mL of 70% (*v*/*v*) ethanol. Calibration curves were obtained by LC–PDA analysis of serial dilutions of the standards prepared in 70% ethanol. The limits of detection (LODs) and quantification (LOQs) were calculated based on the standard deviation (σ) of the response for the lowest calibration level and the slope (S) of the calibration curve, according to the following equations: LOD = 3.3σ/S and LOQ = 10σ/S. Due to the limited availability of authentic standards, semi-quantitative analysis was performed using calibration curves of selected reference compounds. Results were expressed as equivalents of the corresponding reference standard. Luteolin, apigenin, and quercetin derivatives were expressed as luteolin, apigenin, and quercetin equivalents, respectively.

Acacetin derivatives were expressed as apigenin equivalents. N-Coumaroylserotonin hexoside was expressed as *p*-coumaric acid equivalents, whereas lignan derivatives were expressed as ferulic acid equivalents. No molecular-weight correction was applied; therefore, the reported values represent equivalents of the respective reference compounds rather than absolute concentrations of the individual metabolites.

Semi-quantitative analysis was performed using PDA chromatograms recorded at wavelengths selected according to the spectral characteristics of the respective compound classes. Flavonoids were quantified at 360 nm, whereas N-coumaroylserotonin hexoside and lignan derivatives were quantified at 325 nm. The chromatogram recorded at 270 nm was used only for visualization of the overall chromatographic profile and was not used for semi-quantitative calculations.

The chromatographic column and separation conditions were identical to those used for the LC–Q-Orbitrap HRMS method.

#### 4.2.4. Post-Column ABTS Antioxidant Profiling

Chromatographic and antioxidant activity profiles were obtained using an HPLC–PDA system (Agilent Technologies, Wilmington, DE, USA) coupled to a Pinnacle PCX post-column derivatization instrument (Pickering Laboratories Inc., Mountain View, CA, USA) and a UV–Vis detector (Agilent Technologies). The chromatographic column and separation conditions were identical to those used for the LC–HRMS analysis. UV chromatograms prior to derivatization were recorded at 270 nm with the PDA detector. ABTS was dissolved in an aqueous Na_2_S_2_O_8_ solution (2.45 mM) to obtain a final ABTS concentration of 7 mM and kept in the dark at room temperature for at least 6 h to generate the ABTS radical cation. The resulting ABTS radical solution was subsequently diluted with methanol to a working concentration of 1 mM. The column effluent was then directed to the post-column module, where it was mixed with a methanolic ABTS solution delivered at 0.10 mL min^−1^ and passed through a reaction coil (1.0 mL, 130 °C). The resulting ABTS assay profiles were monitored at 734 nm with the UV–Vis detector. The procedure followed Kusznierewicz et al. [60,61] with minor modifications.

### 4.3. Data Analysis

Semi-quantitative data are presented as mean ± standard deviation (SD) of three parallel extractions performed from a pooled lyophilized sample obtained for each harvest time. No inferential statistical tests were applied to compare CT1, CT2, and CT3 because the parallel extractions did not represent independent biological replicates.

Principal component analysis (PCA) and hierarchical clustering analysis (HCA) were performed using MetaboAnalyst 6.0 as exploratory multivariate approaches to visualize differences in metabolite profiles among the three harvest times. For PCA, the QC-corrected peak-area matrix comprising 76 annotated metabolites and nine extracts was log10-transformed and Pareto-scaled prior to analysis. For hierarchical clustering and heatmap visualization, feature-wise standardized peak-area values were used. Hierarchical clustering of both samples and metabolites was performed using Euclidean distance and Ward’s linkage method.

## 5. Conclusions

The available literature on alterations in the metabolite profiles of safflower (*Carthamus tinctorius* L.) young shoots according to varying harvest times is scarce. In the young shoots of safflower, a total of 76 metabolites were tentatively identified. The identified compounds belonged to several major phytochemical groups, including amino acids and related compounds, carbohydrates, other glycosides, phenylpropanoid glycosides, apocarotenoids, hydroxycinnamic acid amides, flavonoids, lignans, and lipids.

Exploratory PCA and hierarchical clustering revealed clear differences in the metabolite profiles of safflower young shoots harvested at 11, 18, and 21 days after sowing. Most of the semi-quantified metabolites showed lower contents at the later harvest times, although distinct temporal patterns were observed for individual metabolites. Because the samples represented successive harvest times under natural greenhouse light conditions, these differences should be interpreted as harvest-time-associated changes rather than effects attributable exclusively to plant developmental stage.

Post-column ABTS profiling indicated that antioxidant activity was associated predominantly with quercetin and luteolin derivatives, while differences in antioxidant response were observed among the other phenolic constituents.

Young shoots of safflower, collected at the optimal stage of development and added to daily meals, can provide a valuable natural source of bioactive compounds. Furthermore, they have the potential to act as a natural preventive strategy against chronic, non-communicable diseases.

## Figures and Tables

**Table 1:** Tentative identification of metabolites detected in safflower (*Carthamus tinctorius* L.) young shoot extracts by LC-HRMS/MS. Metabolite annotations were assigned based on accurate mass measurements, molecular formula prediction, characteristic MS/MS fragmentation patterns, comparison with literature data, and database searches. The table summarizes retention times, molecular formulas, precursor ions, diagnostic product ions, and proposed fragment assignments used for metabolite characterization.

**No.**	**RT [min]**	**Class**	**Tentative Annotation (MSI Level)**	Formula	Precursor Ion				Diagnostic Product Ions		
					Theoretical *m*/*z*	Experimental *m*/*z*	Ion Type	Δm [ppm]	*m*/*z*	Fragment formula	Proposed Fragment Assignment
1	1.61	AA	Spermidine (MSI 2)	C_7_H_19_N_3_	146.16517	146.16544	[M + H]^+^	1.8	72.08157	C_4_H_10_N	Spermidine-derived
									84.08147	C_5_H_10_N	
									112.11240	C_7_H_14_N	
2	1.89	CH	Disaccharide(MSI 3)	C_12_H_22_O_11_	341.10894	341.10861	[M − H]^−^	−1.0	89.02300	C_3_H_5_O_3_	Carbohydrate-derived
									179.05502	C_6_H_11_O_6_	Hexose-derived
									161.04477	C_6_H_9_O_5_	Dehydrated hexose
3	2.19	AA	Phenylalanine (MSI 2)	C_9_H_11_NO_2_	166.08626	166.08651	[M + H]^+^	1.5	103.05474	C_8_H_7_	Phenylalanine-derived
									120.08109	C_8_H_10_N	Phenethylamine-type
									91.05485	C_7_H_7_	Tropylium ion
4	2.39	AA	Leucine/isoleucine (MSI 3)	C_6_H_13_NO_2_	132.10191	132.10224	[M + H]^+^	2.5	86.09709	C_5_H_12_N	Leucine/isoleucine immonium ion
5	2.71	AA	Phenylalanine-related compound (MSI 3)	C_9_H_11_NO_2_	166.08626	166.08663	[M + H]^+^	2.3	103.05476	C_8_H_7_	Phenylalanine-derived
									120.08112	C_8_H_10_N	Phenethylamine-type
									91.05487	C_7_H_7_	Tropylium ion
6	3.47	AA	Tryptophan (MSI 2)	C_11_H_12_N_2_O_2_	205.09715	205.09738	[M + H]^+^	1.1	118.06546	C_8_H_8_N	Indole-derived
									146.06010	C_9_H_8_NO	Indole-carboxaldehyde-type ion
									143.07320	C_10_H_9_N	Deaminated indole-derived
7	4.21	OG	Rengyoxide hexoside (Rengyoside B) (MSI 2)	C_14_H_24_O_8_	321.15439	321.15445	[M + H]^+^	0.2	115.07572	C_6_H_11_O_2_	Rengyoxide—C_2_H_4_O
									123.08061	C_8_H_11_O	Rengyoxide—2H_2_O
									141.09108	C_8_H_13_O_2_	Rengyoxide—H_2_O
8	5.08	PPG	Syringin (MSI 2)	C_17_H_24_O_9_	371.13476	371.13481	[M − H]^−^	0.1	195.06570	C_10_H_11_O_4_	Sinapyl alcohol-derived
									209.08113	C_11_H_13_O_4_	Sinapyl alcohol aglycone
9	5.42	PPG	Dihydrosyringin (MSI 2)	C_17_H_26_O_9_	373.15041	373.15051	[M − H]^−^	0.3	211.09731	C_11_H_15_O_4_	Dihydrosinapyl alcohol aglycone
									196.07390	C_10_H_12_O_4_	Dihydrosinapyl alcohol-derived
10	6.33	FLV	Apigenin-*C*-dihexoside (MSI 3)	C_27_H_30_O_15_	593.15119	593.15142	[M − H]^−^	0.4	473.10913	C_23_H_21_O_11_	C-glycoside cleavage
									353.06677	C_19_H_13_O_7_	Apigenin + C_4_H_4_O_2_
									383.07733	C_20_H_15_O_8_	Apigenin + C_5_H**_7_**O_3_
11	6.39	OG	Thalictoside (MSI 2)	C_14_H_19_NO_8_	328.10379	328.10384	[M − H]^−^	0.1	60.00773	CH_2_NO_2_	Nitroethyl-derived
									166.0504	C_8_H_8_NO_3_	Nitroethylphenol aglycone
12	6.88	LIG	Syringaresinol-O- hexoside (MSI 3)	C_28_H_36_O_13_	579.20832	579.20888	[M − H]^−^	1.0	399.14505	C_22_H_23_O_7_	Syringaresinol—H_2_O
									417.15585	C_22_H_25_O_8_	Syringaresinol aglycone
13	6.88	APC	Roseoside (MSI 2)	C_19_H_30_O_8_	387.20135	387.20117	[M + H]^+^	−0.4	207.13805	C_13_H_19_O_2_	Vomifoliol—H_2_O
									189.12733	C_13_H_17_O	Vomifoliol—2H_2_O
14	7.26	FLV	Apigenin-*C*-pentosyl-*C*-hexoside (MSI 3)	C_26_H_28_O_14_	563.14063	563.14117	[M − H]^−^	1.0	353.06693	C_19_H_13_O_7_	Apigenin + C_4_H_4_O_2_
									473.10919	C_23_H_21_O_11_	C-glycoside cleavage
									383.07730	C_20_H_15_O_8_	Apigenin + C_5_H**_7_**O_3_
									443.09863	C_22_H_19_O_10_	C-glycoside cleavage
									503.11996	C_24_H_23_O_12_	C-glycoside cleavage
15	7.85	LIG	Olivil hexoside (MSI 3)	C_26_H_34_O_12_	537.19775	537.19781	[M − H]^−^	0.1	191.07065	C_11_H_11_O_3_	Olivil-derived
									345.13446	C_19_H_21_O_6_	Olivil—CH_2_O
									209.08145	C_11_H_13_O_4_	Olivil-derived
									375.14444	C_20_H_23_O_7_	Olivil aglycone
16	8.28	HCAA	N-Coumaroylserotonin hexoside I (MSI 3)	C_25_H_28_N_2_O_8_	483.17729	483.17764	[M − H]^−^	0.7	321.12457	C_19_H_17_N_2_O_3_	N-Coumaroylserotonin aglycone
									119.04894	C_8_H_7_O	Coumaroyl-derived
									201.06635	C_11_H_9_N_2_O_2_	Serotonin-derived
									145.02856	C_9_H_5_O_2_	Coumaroyl-derived
									175.08699	C_10_H_11_N_2_O	Serotonin-derived
17	9.00	HCAA	N-Feruloylserotonin hexoside I (MSI 3)	C_26_H_30_N_2_O_9_	513.18786	513.18829	[M − H]^−^	0.8	351.13516	C_20_H_19_N_2_O_4_	N-Feruloylserotonin aglycone
									149.05972	C_9_H_9_O_2_	Feruloyl-derived
									201.06644	C_11_H_9_N_2_O_2_	Serotonin-derived
									336.11179	C_19_H_16_N_2_O_4_	N-Feruloylserotonin—CH_3_
									135.04408	C_8_H_7_O_2_	Feruloyl-derived
18	9.18	FLV	Apigenin-*C*-hexoside (MSI 3)	C_21_H_20_O_10_	431.09837	431.09861	[M − H]^−^	0.6	311.05646	C_17_H_11_O_6_	C-glycoside cleavage
									283.06128	C_16_H_11_O_5_	
									341.06659	C_18_H_13_O_7_	
19	9.37	HCAA	N-Coumaroylserotonin hexoside II (MSI 3)	C_25_H_28_N_2_O_8_	483.17729	483.17749	[M − H]^−^	0.4	321.12457	C_19_H_17_N_2_O_3_	N-Coumaroylserotonin aglycone
									119.04897	C_8_H_7_O	Coumaroyl-derived
									201.06630	C_11_H_9_N_2_O_2_	Serotonin-derived
									145.02847	C_9_H_5_O_2_	Coumaroyl-derived
									175.08667	C_10_H_11_N_2_O	Serotonin-derived
20	9.40	FLV	Quercetin-*O*-hexoside (MSI 3)	C_21_H_20_O_12_	463.08820	463.08850	[M − H]^−^	0.6	301.03537	C_15_H_9_O_7_	Quercetin aglycone
21	9.95	FLV	Luteolin-*O*-hexoside I (MSI 3)	C_21_H_20_O_11_	447.09329	447.09353	[M − H]^−^	0.5	285.04041	C_15_H_9_O_6_	Luteolin aglycone
22	10.26	HCAA	N-Feruloylserotonin hexoside II (MSI 3)	C_26_H_30_N_2_O_9_	513.18786	513.18823	[M − H]^−^	0.7	351.13519	C_20_H_19_N_2_O_4_	N-Feruloylserotonin aglycone
									149.05975	C_9_H_9_O_2_	Feruloyl-derived
									336.11166	C_19_H_16_N_2_O_4_	N-Feruloylserotonin—CH_3_
									201.06622	C_11_H_9_N_2_O_2_	Serotonin-derived
									135.04399	C_8_H_7_O_2_	Feruloyl-derived
23	11.32	HCAA	N-Coumaroylserotonin malonylhexoside (MSI 3)	C_28_H_30_N_2_O_11_	569.17768	569.17824	[M − H]^−^	1.0	321.12460	C_19_H_17_N_2_O_3_	N-Coumaroylserotonin aglycone
									119.04898	C_8_H_7_O	Coumaroyl-derived
									465.16776	C_25_H_25_N_2_O_7_	N-Coumaroylserotonin hexoside—H_2_O
									483.17776	C_25_H_27_N_2_O_8_	N-Coumaroylserotonin hexoside
									525.18756	C_27_H_29_N_2_O_9_	Precursor—CO_2_
24	11.48	FLV	Apigenin-*O*-pentosyl-*O*-hexoside (MSI 3)	C_26_H_28_O_14_	563.14063	563.140869	[M − H]^−^	0.4	269.04565	C_15_H_9_O_5_	Apigenin aglycone
									431.09872	C_21_H_19_O_10_	Apigenin-*O*-hexoside
25	11.53	FLV	Quercetin-*O*-acetylhexoside-*O*- pentoside (MSI 3)	C_28_H_30_O_17_	637.14102	637.14166	[M − H]^−^	1.0	301.03531	C_15_H_9_O_7_	Quercetin aglycone
									505.09955	C_23_H_21_O_13_	Quercetin-*O*-acetylhexoside
26	11.74	FLV	Quercetin-*O*-malonylhexoside (MSI 3)	C_24_H_22_O_15_	549.08859	549.08898	[M − H]^−^	0.7	301.03558	C_15_H_9_O_7_	Quercetin aglycone
									505.09967	C_23_H_21_O_13_	Precursor—CO_2_
27	11.84	FLV	Apigenin-*O*-hexoside (MSI 3)	C_21_H_20_O_10_	431.09837	431.09860	[M − H]^−^	0.5	269.04456	C_15_H_9_O_5_	Apigenin aglycone
28	11.89	HCAA	N-Coumaroylserotonin I (MSI 3)	C_19_H_18_N_2_O_3_	321.12447	321.12450	[M − H]^−^	0.1	119.04898	C_8_H_7_O	Coumaroyl-derived
									175.08682	C_10_H_11_N_2_O	Serotonin-derived
									145.02843	C_9_H_5_O_2_	Coumaroyl-derived
29	12.04	FLV	Luteolin-*O*-pentosyl-*O*-malonylhexoside (MSI 3)	C_29_H_30_O_18_	665.13594	665.13666	[M − H]^−^	1.1	621.14655	C_28_H_29_O_16_	Precursor—CO_2_
									285.04025	C_15_H_9_O_6_	Luteolin aglycone
									489.10425	C_23_H_21_O_12_	Decarboxylated luteolin glycoside
									579.13483	C_26_H_27_O_15_	Luteolin pentosylhexoside
30	12.06	FLV	Apigenin-*O*-glucuronide (MSI 3)	C_21_H_18_O_11_	445.07764	445.07790	[M − H]^−^	0.6	269.04572	C_15_H_9_O_5_	Apigenin aglycone
31	12.28	FLV	Luteolin-*O*-malonylhexoside (MSI 3)	C_24_H_22_O_14_	533.09368	533.09411	[M − H]^−^	0.8	489.10428	C_23_H_21_O_12_	Precursor—CO_2_
									285.04047	C_15_H_9_O_6_	Luteolin aglycone
									327.05130	C_17_H_11_O_7_	Luteolin-derived
									447.09113	C_21_H_19_O_11_	Luteolin-*O*-hexoside
32	12.32	LIG	Matairesinol hexoside-pentoside (MSI 3)	C_31_H_40_O_15_	651.22944	651.22979	[M − H]^−^	0.5	357.13461	C_20_H_21_O_6_	Matairesinol aglycone
									343.11984	C_19_H_19_O_6_	Matairesinol-derived
33	12.34	LIG	Hydrated trachelogenin hexoside (MSI 3)	C_27_H_36_O_13_	567.20832	567.20861	[M − H]^−^	0.5	357.13455	C_20_H_21_O_6_	Trachelogenin-derived fragment (−CH_2_O)
									387.14517	C_21_H_23_O_7_	Trachelogenin
									329.13965	C_19_H_21_O_5_	Trachelogenin-derived
									405.15527	C_21_H_25_O_8_	Hydrated trachelogenin aglycone
34	12.54	LIG	Lignan dihexoside (MSI 3)	C_32_H_42_O_16_	681.24001	681.24017	[M − H]^−^	0.2	651.23029	C_31_H_39_O_15_	Precursor—CH_2_O
									357.13431	C_20_H_21_O_6_	Aglycone
									329.13968	C_19_H_21_O_5_	Aglycone-derived fragment
35	12.66	FLV	Luteolin-*O*-hexoside II (MSI 3)	C_21_H_20_O_11_	447.09329	447.09361	[M − H]^−^	0.7	285.04050	C_15_H_9_O_6_	Luteolin aglycone
36	12.87	LIG	Matairesinol-*O*-hexoside (MSI 3)	C_26_H_32_O_11_	519.18719	519.18756	[M − H]^−^	0.7	357.13464	C_20_H_21_O_6_	Matairesinol aglycone
37	13.10	HCAA	N-Coumaroylserotonin II (MSI 3)	C_19_H_18_N_2_O_3_	321.12447	321.12451	[M − H]^−^	0.1	119.04899	C_8_H_7_O	Coumaroyl-derived
									175.08684	C_10_H_11_N_2_O	Serotonin-derived
									145.02850	C_9_H_5_O_2_	Coumaroyl-derived
38	13.26	LIG	Tracheloside (MSI 2)	C_27_H_34_O_12_	549.19775	549.19825	[M − H]^−^	0.9	387.14529	C_21_H_23_O_7_	Trachelogenin aglycone
									329.13962	C_19_H_21_O_5_	Trachelogenin-derived
39	13.77	FLV	Apigenin-*O*-malonylhexoside-*O*-pentoside (MSI 3)	C_29_H_30_O_17_	649.14103	649.14151	[M − H]^−^	0.7	605.15198	C_28_H_29_O_15_	Precursor—CO_2_
									269.04568	C_15_H_9_O_5_	Apigenin aglycone
									563.14105	C_26_H_27_O_14_	Apigenin-*O*-pentoside hexoside
40	14.10	HCAA	N-Feruloylserotonin (MSI 2)	C_20_H_20_N_2_O_4_	351.13503	351.13511	[M − H]^−^	0.2	149.05566	C_9_H_9_O_2_	Feruloyl-derived
									135.04414	C_8_H_7_O_2_	Feruloyl-derived
									175.08678	C_10_H_11_N_2_O	Serotonin-derived
41	14.24	FLV	Apigenin-*O*-malonylhexoside (MSI 3)	C_24_H_22_O_13_	517.09877	517.09910	[M − H]^−^	0.6	269.04565	C_15_H_9_O_5_	Apigenin aglycone
									431.09793	C_21_H_19_O_10_	Apigenin-*O*-hexoside
42	14.24	FLV	Quercetin-*O*-acetylhexoside (MSI 3)	C_23_H_22_O_13_	505.09877	505.09911	[M − H]^−^	0.7	301.03549	C_15_H_9_O_7_	Quercetin aglycone
43	14.45	FLV	Luteolin-*O*-pentosyl-*O*-acetylhexoside (MSI 3)	C_28_H_30_O_16_	621.14611	621.14666	[M − H]^−^	0.9	285.04034	C_15_H_9_O_6_	Luteolin aglycone
									489.10355	C_23_H_21_O_12_	Luteolin-*O*-acetylhexoside
44	14.73	FLV	Luteolin-*O*-acetylhexoside (MSI 3)	C_23_H_22_O_12_	489.10385	489.10412	[M − H]^−^	0.5	285.04047	C_15_H_9_O_6_	Luteolin aglycone
									327.05170	C_17_H_11_O_7_	Luteolin-derived
45	15.10	LIG	Methylated matairesinol hexoside-pentoside	C_32_H_42_O_15_	665.24509	665.24481	[M − H]^−^	−0.4	371.15018	C_21_H_23_O_6_	Methylated matairesinol aglycone
									357.13422	C_20_H_21_O_6_	Matairesinol
46	15.46	LIP	Steroidal glycoside with hexose and acetylhexose (MSI 3)	C_35_H_54_O_14_	699.35864	699.35838	[M + H]^+^	−0.4	333.24219	C_21_H_33_O_3_	Steroidal aglycone
									297.22113	C_21_H_29_O	Steroidal aglycone − 2H_2_O
									315.23178	C_21_H_31_O_2_	Steroidal aglycone − H_2_O
									205.07072	C_8_H_13_O_6_	Acetylhexose
47	15.74	FLV	Luteolin (MSI 2)	C_15_H_10_O_6_	285.04046	285.04046	[M − H]^−^	0.0	133.02837	C_8_H_5_O_2_	RDA fragment
									151.00266	C_7_H_3_O_4_	
48	15.89	FLV	Quercetin (MSI 2)	C_15_H_10_O_7_	301.03538	301.03511	[M − H]^−^	−0.9	151.00266	C_7_H_3_O_4_	RDA fragment
49	16.44	FLV	Acacetin-*O*-pentosyl-*O*-glucuronide (MSI 3)	C_27_H_28_O_15_	591.13555	591.13598	[M − H]^−^	0.7	283.06125	C_16_H_11_O_5_	Acacetin aglycone
									307.06741	C_11_H_15_O_10_	Sugar moiety-derived
50	16.65	LIG	Matairesinol-related vanilloyl hexoside-pentoside (MSI 3)	C_39_H_46_O_18_	801.26114	801.26196	[M − H]^−^	1.0	633.21954	C_31_H_37_O_14_	Matairesinol glycoside-derived
									167.03404	C_8_H_7_O_4_	Vanilloyl-derived
									357.13440	C_20_H_21_O_6_	Matairesinol aglycone
51	17.61	FLV	Acacetin-*O*-glucuronide (MSI 3)	C_22_H_20_O_11_	459.09329	459.09362	[M − H]^−^	0.7	283.06125	C_16_H_11_O_5_	Acacetin aglycone
52	17.93	LIG	Matairesinol sinapoyl hexoside-pentoside (MSI 3)	C_42_H_50_O_19_	857.28736	857.28796	[M − H]^−^	0.7	193.04993	C_10_H_9_O_4_	Sinapoyl-derived
									633.21960	C_31_H_37_O_14_	Matairesinol glycoside-derived
									357.13443	C_20_H_21_O_6_	Matairesinol aglycone
53	18.41	FLV	Apigenin (MSI 2)	C_15_H_10_O_5_	269.04555	269.04549	[M − H]^−^	−0.2	117.03333	C_8_H_5_O	RDA fragment
									151.00266	C_7_H_3_O_4_	
54	18.61	FLV	Acacetin sinapoyl glucuronide-pentoside (MSI 3)	C_38_H_38_O_19_	797.19346	797.19446	[M − H]^−^	1.3	283.06125	C_16_H_11_O_5_	Acacetin aglycone
									223.06070	C_11_H_11_O_5_	Sinapoyl-derived
									513.12506	C_22_H_25_O_14_	Sugar moiety-derived
55	18.86	LIG	Trachelogenin (MSI 2)	C_21_H_24_O_7_	387.14493	387.14501	[M − H]^−^	0.2	99.00741	C_4_H_3_O_3_	Lactone-derived
									193.08630	C_11_H_13_O_3_	Trachelogenin-derived
									136.05185	C_8_H_8_O_2_	Trachelogenin-derived
56	20.93	FLV	Acylated luteolin-*O*-hexoside (MSI 3)	C_32_H_30_O_14_	637.15628	637.15688	[M − H]^−^	0.9	475.10373	C_26_H_19_O_9_	Acylated luteolin aglycone
									284.03226	C_15_H_8_O_6_	Luteolin derived
57	21.09	LIP	MGMG(18:3;O) (MSI 3)	C_27_H_46_O_10_	575.30730	575.30774	[M+HCOO]^−^	0.8	293.2124	C_18_H_29_O_3_	FA(18:3;O)
									253.0928	C_9_H_17_O_8_	Monogalactosylglycerol
									161.04453	C_6_H_9_O_5_	Hexose-derived
58	21.23	FLV	Acacetin (MSI 2)	C_16_H_12_O_5_	283.06120	283.06112	[M − H]^−^	−0.3	268.03784	C_15_H_8_O_5_	Precursor—CH_3_
									240.04221	C_14_H_8_O_4_	Precursor—CO—CH_3_
									151.00262	C_7_H_3_O_4_	RDA fragment
59	21.63	LIP	FA(18:5;O2), putative (MSI 3)	C_18_H_26_O_4_	305.17583	305.17587	[M − H]^−^	0.1	135.08043	C_9_H_11_O	Oxylipin-derived
60	22.31	LIP	DGMG(18:3) I (MSI 3)	C_33_H_56_O_14_	675.35973	675. 36049	[M − H]^−^	1.1	397.13528	C_15_H_25_O_12_	Digalactosylglycerol
									277.21732	C_18_H_29_O_2_	FA(18:3)
61	22.54	LIP	DGMG(18:3) II (MSI 3)	C_33_H_56_O_14_	675.35973	675.36043	[M − H]^−^	1.0	397.13528	C_15_H_25_O_12_	Digalactosylglycerol
									277.21744	C_18_H_29_O_2_	FA(18:3)
62	23.36	LIP	FA(18:3;O) (MSI 3)	C_18_H_30_O_3_	293.21222	293.21211	[M − H]^−^	−0.4	275.20242	C_18_H_27_O_2_	Precursor—H_2_O
									235.16934	C_15_H_23_O_2_	FA(18:3;O)-derived
63	23.45	LIP	MGMG(18:3) I (MSI 3)	C_27_H_46_O_9_	559.31239	559.31262	[M+HCOO]^−^	0.4	277.21735	C_18_H_29_O_2_	FA(18:3)
									253.09283	C_9_H_17_O_8_	Monogalactosylglycerol
									161.04443	C_6_H_9_O_5_	Hexose-derived
64	23.66	LIP	SQDG(34:3) I (MSI 3)	C_43_H_76_O_12_S	815.49848	815.49884	[M − H]^−^	0.5	225.00777	C_6_H_9_O_7_S	Sulfoquinovosyl
65	23.67	LIP	MGMG(18:3) II (MSI 3)	C_27_H_46_O_9_	559.31239	559.31261	[M+HCOO]^−^	0.4	277.21732	C_18_H_29_O_2_	FA(18:3)
									253.09279	C_9_H_17_O_8_	Monogalactosylglycerol
									161.04472	C_6_H_9_O_5_	Hexose-derived
66	23.97	LIP	SQDG(36:6) (MSI 3)	C_45_H_74_O_12_S	837.48283	837.48297	[M − H]^−^	0.2	225.00752	C_6_H_9_O_7_S	Sulfoquinovosyl
67	23.98	LIP	PE(16:0_16:1) (MSI 3)	C_37_H_72_NO_8_P	688.49228	688.49280	[M − H]^−^	0.8	253.21727	C_16_H_29_O_2_	FA(16:1)
									255.23285	C_16_H_31_O_2_	FA(16:0)
									140.01073	C_2_H_7_NO_4_P	Phosphoethanolamine
68	24.03	LIP	FA(18:2;O) (MSI 3)	C_18_H_32_O_3_	295.22787	295.22787	[M − H]^−^	0.0	171.10179	C_9_H_15_O_3_	Oxylipin-derived
									277.21713	C_18_H_29_O_2_	Precursor—H_2_O
									195.13866	C_12_H_19_O_2_	Oxylipin-derived
69	25.92	LIP	SQMG(18:3) (MSI 3)	C_27_H_46_O_11_S	577.26881	577.26923	[M − H]^−^	0.7	225.00703	C_6_H_9_O_7_S	Sulfoquinovosyl
									299.04510	C_9_H_15_O_9_S	Sulfoquinovosylglycerol
70	26.11	LIP	SQDG(34:3) II (MSI 3)	C_43_H_76_O_12_S	815.49848	815.49921	[M − H]^−^	0.9	225.00743	C_6_H_9_O_7_S	Sulfoquinovosyl
71	26.65	LIP	DGDG(18:3_18:3;O) (MSI 3)	C_51_H_84_O_16_	997.57414	997.57449	[M + HCOO]^−^	0.4	293.21237	C_18_H_29_O_3_	FA(18:3;O)
									397.13522	C_15_H_25_O_12_	Digalactosylglycerol
									277.21719	C_18_H_29_O_2_	FA(18:3)
72	26.78	LIP	SQDG(34:3) III (MSI 3)	C_43_H_76_O_12_S	815.49848	815.49924	[M − H]^−^	0.9	225.00700	C_6_H_9_O_7_S	Sulfoquinovosyl
73	27.34	LIP	SQDG(34:3) IV (MSI 3)	C_43_H_76_O_12_S	815.49848	815.49908	[M − H]^−^	0.7	225.00627	C_6_H_9_O_7_S	Sulfoquinovosyl
74	27.50	LIP	MGDG(18:3_18:3;O) (MSI 3)	C_45_H_74_O_11_	835.52131	835.52212	[M+HCOO]^−^	1.0	277.21729	C_18_H_29_O_2_	FA(18:3)
									293.21237	C_18_H_29_O_3_	FA(18:3;O)
									253.09276	C_9_H_17_O_8_	Monogalactosylglycerol
75	27.60	LIP	HexCer(34:1;O3) (MSI 3)	C_40_H_77_NO_9_	714.55256	714. 55286	[M − H]^−^	0.4	534.48975	C_34_H_64_NO_3_	Ceramide—H_2_O
									179.05524	C_6_H_11_O_6_	Hexose-derived
									552.49982	C_34_H_66_NO_4_	Ceramide aglycone
76	29.62	LIP	MGDG(18:3_18:3) (MSI 3)	C_45_H_74_O_10_	773.52092	773.52168	[M − H]^−^	1.0	277.21732	C_18_H_29_O_2_	FA(18:3)
									253.09247	C_9_H_17_O_8_	Monogalactosylglycerol
									235.08212	C_9_H_15_O_7_	Monogalactosylglycerol − H_2_O
									161.04411	C_6_H_9_O_5_	Hexose-derived

MSI annotation confidence levels are indicated in parentheses after each metabolite annotation: MSI 2, putatively annotated compound; MSI 3, putatively characterized compound class, according to the Metabolomics Standards Initiative criteria proposed by Sumner et al. [19]. No compounds were assigned to MSI Level 1 because authentic reference standards were not used for confirmatory identification based on matching retention time and MS/MS spectra under identical analytical conditions. Roman numerals (I–IV) denote chromatographically resolved features with the same general annotation and do not imply assignment of specific positional or structural isomers. Lipid nomenclature follows the recommendations of Liebisch et al. [20] and reflects the level of structural information supported by the available accurate-mass and MS/MS data; fatty-acyl composition and other structural details were included only when supported by diagnostic fragment ions. Abbreviations: AcHex, acetylhexose; AA, amino acid; APC, apocarotenoid; CH, carbohydrate; DGDG, digalactosyldiacylglycerol; DGMG, digalactosylmonoacylglycerol; FA, fatty acid; FLV, flavonoid; HCAA, hydroxycinnamic acid amide; Hex, hexose; HexCer, hexosylceramide; LIG, lignan; LIP, lipid; MGDG, monogalactosyldiacylglycerol; MGMG, monogalactosylmonoacylglycerol; OG, other glycoside; PE, phosphatidylethanolamine; Pent, pentose; PPG, phenylpropanoid glycoside; RDA, retro-Diels–Alder fragmentation; SQDG, sulfoquinovosyldiacylglycerol; SQMG, sulfoquinovosylmonoacylglycerol.

**Table 2:** Semi-quantitative contents of the major metabolites identified in safflower (*Carthamus tinctorius* L.) young shoot extracts (CT1–CT3) and their antioxidant activity evaluated by the post-column ABTS assay. Compound contents are expressed as mg equivalents per g of dry weight (mg eq./g DW) using calibration curves of selected reference standards, as specified below the table. Antioxidant activity was assigned based on the presence (+) or absence (−) of negative peaks in the post-column ABTS chromatogram.

Peak No.	Name	Content (mg eq./g DW)			Antioxidant Activity
		CT1	CT2	CT3	
10	Apigenin-*C*-dihexoside (1)	0.2651 ± 0.0031 †	0.2174 ± 0.0026 †	0.2654 ± 0.0026 †	**−**
16	N-Coumaroylserotonin hexoside (2)	0.081 ± 0.013	0.0574 ± 0.0052	0.0517 ± 0.0014	**+**
20	Quercetin-*O*-hexoside (3)	1.538 ± 0.015 †	0.839 ± 0.018 †	0.6599 ± 0.0031 †	**+**
21	Luteolin-*O*-hexoside (4)	4.422 ± 0.045	2.865 ± 0.020	2.294 ± 0.015	**+**
26	Quercetin-*O*-malonylhexoside (3)	2.434 ± 0.042	1.211 ± 0.029	0.7991 ± 0.0057	**+**
31	Luteolin-*O*-malonylhexoside (4)	0.594 ± 0.014 †	0.4587 ± 0.0036 †	0.3135 ± 0.0012 †	**+**
32	Matairesinol hexoside-pentoside (5)	0.1491 ±0.0036 †	0.09258 ± 0.00066 †	0.05424 ± 0.00037 †	**−**
38	Tracheloside (5)	0.5319 ± 0.0046	0.3388 ± 0.0026	0.24356 ± 0.00068	**−**
44	Luteolin-*O*-acetylhexoside (4)	0.6580 ± 0.0074	0.4293 ± 0.0040	0.3757 ± 0.0010	**+**
49	Acacetin-*O*-glucuronide-pentoside (1)	0.8518 ± 0.0084	0.5880 ± 0.0044	0.34591 ± 0.00059	**−**
51	Acacetin-*O*-glucuronide (1)	3.659 ± 0.037	2.751 ± 0.013	1.268 ± 0.089	**−**
54	Acacetin sinapoyl glucuronide-pentoside (1)	0.8014 ± 0.0070	0.5181 ± 0.0052	0.2259 ± 0.0010	**+**
55	Trachelogenin (5)	0.1775 ± 0.0024	0.1581 ± 0.0010	0.1106 ± 0.0029	**−**

Contents were semi-quantified as equivalents of the following reference compounds: (1) apigenin; (2) p-coumaric acid; (3) quercetin; (4) luteolin; and (5) ferulic acid. Results are expressed as mg equivalents per g dry weight (mg eq./g DW) and presented as mean ± SD of three parallel extractions performed from the same pooled lyophilized sample for each harvest time. † Peaks 10, 20, 31, and 32 were not fully chromatographically resolved from co-eluting metabolites (10/11, 19/20, and 31–33, respectively). Consequently, the corresponding PDA peak areas may include contributions from the co-eluting compounds. The reported values should therefore be regarded as approximate semi-quantitative estimates of the respective unresolved chromatographic signals rather than compound-specific contents and were not used to establish abundance rankings among individual metabolites. Calibration parameters were as follows: apigenin, linear range 6–70 µg/mL, y = 13967x + 4, R^2^ = 0.999, LOD = 0.6 µg/mL, LOQ = 1.8 µg/mL; p-coumaric acid, linear range 7–94 µg/mL, y = 33374x − 24, R^2^ = 0.999, LOD = 0.6 µg/mL, LOQ = 1.8 µg/mL; quercetin, linear range 7–87 µg/mL, y = 14674x − 20, R^2^ = 0.999, LOD = 0.3 µg/mL, LOQ = 1.0 µg/mL; luteolin, linear range 6–80 µg/mL, y = 15989x − 20, R^2^ = 0.999, LOD = 0.3 µg/mL, LOQ = 0.8 µg/mL; and ferulic acid, linear range 8–100 µg/mL, y = 36285x − 29, R^2^ = 0.999, LOD = 0.6 µg/mL, LOQ = 1.9 µg/mL.

## Data Availability

The LC–HRMS metabolomics data generated in this study have been deposited in the MetaboLights repository under the study identifier MTBLS16150.
